# Complex network-based classification of radiographic images for COVID-19 diagnosis

**DOI:** 10.1371/journal.pone.0290968

**Published:** 2023-09-01

**Authors:** Weiguang Liu, Rafael Delalibera Rodrigues, Jianglong Yan, Yu-tao Zhu, Everson José de Freitas Pereira, Gen Li, Qiusheng Zheng, Liang Zhao

**Affiliations:** 1 School of Computer Science, Zhongyuan University of Technology, ZhengZhou, China; 2 Institute of Mathematics and Computer Science (ICMC), University of São Paulo (USP), São Carlos, Brazil; 3 China Branch of BRICS Institute of Future Networks, ShenZhen, China; 4 Henan Key Laboratory on Public Opinion Intelligent Analysis, Zhongyuan University of Technology, ZhengZhou, China; 5 Department of Computing and Mathematics, FFCLRP, University of São Paulo (USP), Ribeirão Preto, Brazil; Nanjing University of Information Science and Technology, CHINA

## Abstract

In this work, we present a network-based technique for chest X-ray image classification to help the diagnosis and prognosis of patients with COVID-19. From visual inspection, we perceive that healthy and COVID-19 chest radiographic images present different levels of geometric complexity. Therefore, we apply fractal dimension and quadtree as feature extractors to characterize such differences. Moreover, real-world datasets often present complex patterns, which are hardly handled by only the physical features of the data (such as similarity, distance, or distribution). This issue is addressed by complex networks, which are suitable tools for characterizing data patterns and capturing spatial, topological, and functional relationships in data. Specifically, we propose a new approach combining complexity measures and complex networks to provide a modified high-level classification technique to be applied to COVID-19 chest radiographic image classification. The computational results on the Kaggle COVID-19 Radiography Database show that the proposed method can obtain high classification accuracy on X-ray images, being competitive with state-of-the-art classification techniques. Lastly, a set of network measures is evaluated according to their potential in distinguishing the network classes, which resulted in the choice of communicability measure. We expect that the present work will make significant contributions to machine learning at the semantic level and to combat COVID-19.

## Introduction

In late 2019, a viral respiratory disease outbreak emerged, named “coronavirus disease 2019” (COVID-19), caused by a new type of coronavirus called SARS-CoV-2 (Severe Acute Respiratory Syndrome Coronavirus 2) [1–4]. It presents unique virological features, which boosts its transmission efficiency, e.g., presenting a high viral load during the first week of symptoms, which increases the pharyngeal virus shedding [3]. This feature drastically reduces the interval between symptoms onset and the peak of infectivity and in conjunction with the high proportion of mild illness facilitates undetected transmission [1, 5], resulting in a quite high R-naught (basic reproduction number) [6–8]. Thus, the high transmission efficiency of the virus and the abundance of international travel rapidly turned the COVID-19 outbreak into a worldwide pandemic. Therefore, containment measures are very important to reduce COVID-19 spreading, requiring quick and precise testing for timely patient identification.

The early diagnosis of COVID-19 patients is of utmost importance, not only for the patient’s prognosis but also for optimizing hospital resources in response to the COVID-19 pandemic. Efficiency is critical to avoid a crisis in the healthcare system and, consequently, an increase in the number of deaths [9]. In a pandemic, the shortage of resources is practically unavoidable, thus any available resource and tool that can help should be employed to combat the COVID-19 public health emergency. In this context, medical imaging techniques play a crucial role in accurately diagnosing and assessing chest involvement in COVID-19 patients. While Magnetic Resonance Imaging (MRI) and Computed Tomography (CT) imaging techniques provide higher resolution, they are costlier and less accessible compared to X-ray imaging. This is an important issue for public health organizations and low-income people. Therefore, chest X-ray images are a more encompassing technique in the context of COVID-19.

The field of COVID-19 chest X-ray image processing has seen significant advancements with various studies focusing on image segmentation and classification. Most of these works rely heavily on deep learning techniques, such as Convolutional Neural Networks (CNNs) and transfer learning, to achieve high accuracy in detecting COVID-19 and other diseases. However, these deep learning techniques often demand substantial computational resources and lack explicit interpretability in their learning results.

Moreover, traditional data classification algorithms rely only on physical characteristics extracted from data, such as similarity, distance, or distribution, to determine the representation of data classes. These methods are called *low-level classification* and are particularly susceptible to errors when dealing with complex problems, e.g., distinct contexts, overlapping classes, etc. Oppositely, human beings intuitively deal with complex scenarios, classifying objects at an organizational and semantic level, taking into consideration pattern recognition. The computational methods that take into consideration not only physical aspects of data but also pattern formation are called *high-level classification* [10].

Distinctively, high-level classification techniques require a singular fundamental data structure to support an enhanced representation of data classes: complex networks. This representation arises from the complex networks’ research, which deals with many real systems that naturally behave as a network or benefit a lot from an abstract representation in the network form, also known as Network Science [11]. Complex networks refer to large-scale graphs with nontrivial connection patterns [11–16]. This type of network is a suitable tool for characterizing data patterns due to its ability to capture spatial, topological, and functional relationships in data. The interconnections between nodes in a network naturally allow for the identification of patterns, requiring only some suitably defined measures; thus producing an intrinsic high-level classifier.

From this perspective, by analyzing healthy and COVID-19 lung X-ray images, it is possible to see that both classes present visually distinguishable patterns, such as the formation of filaments on COVID-19 images that spread through the X-ray image as an opacity texture. Such a finding implies that the two classes of images present different geometrical complexity. For this reason, we inspect two complexity measures to characterize these differences: fractal dimensions [17, 18] and quadtree [19].

Thus, in this work, we seek to identify automatically the diagnosis of COVID-19 through the analysis of chest X-ray images, using a new approach that combines complexity measures and complex networks to provide a modified high-level classification technique. In the proposed scheme, the complexity measures are employed in the feature extraction phase, extracting characteristics of the images that are relevant to the applied problem, in this specific case, those that help to distinguish the normal and COVID-19 classes. In a high-level classifier, this step provides the data for constructing the network representations for each class. This distinct approach emphasizes pattern formation within the data rather than relying solely on physical features, providing a straightforward and explainable approach to data classification. By leveraging complexity measures and complex networks, our method offers a unique perspective on COVID-19 chest X-ray image classification, combining accuracy with explainability.

Our work is inspired by the original idea of high-level classification, proposed in [10, 20] and extended in [21, 22]. However, our modified high-level technique is not hybrid, not requiring an applied combination with low-level classifiers, in contrast to [10, 20]. Besides that, although the works [21, 22] also eliminate the need for the low-level classification part, these works demand the comparison of diverse network measures, requiring the optimization of various hardly tunable parameters. For this reason, we introduce a modified high-level classification technique using only one network measure, the communicability measure [23], which eliminates those parameter calibration tasks.

Finally, our work aims to analyze chest X-ray images to assist in the diagnosis and prognosis of patients with COVID-19. We employ a pair of complexity measures in conjunction with a modified high-level classification technique. The technique captures and explores the complex topological properties of the networks built for each of the classes from the input data, performing the classification of healthy and COVID-19 X-ray images according to the conformity of the testing sample to the network structure of each class, where its insertion causes the least variation of the network measure under consideration. The experimental results show that the proposed method achieves high accuracy in the classification task, being competitive with state-of-the-art techniques. The primary contributions of this paper are summarized as follows:

1. In this paper, we propose a new high-level data classification technique capable of classifying data samples according to the pattern formation of each class instead of physical features, such as distance, similarity, or distributions. Moreover, we find that complex networks are suitable solutions to characterize data patterns.
2. Our hypothesis is that the COVID-19 images, although present high variations, may share some hidden patterns, therefore, the high-level technique for COVID-19 image classification is a suitable choice. The high classification precision obtained by our method in the simulations using artificial and real datasets confirms such a hypothesis.
3. State-of-the-art classification techniques, such as deep learning techniques, require massive computing power and do not provide a logical explanation of the learning results. On the contrary, our method, although requires large memory space for a large network, presents a straightforward and explainable way for data classification and requires the tuning of only a single parameter *k*, which serves to network construction from the original data.
4. Instead of using statistical measures, we apply fractal dimension and quadtree measures to characterize complexity levels between different classes of images.

## Related works

Hereupon, many studies have achieved great successes in identifying COVID-19 through chest image processing for computer-aided medical diagnosis [24–26]. These can differ in several aspects, such as the encompassed stage or stages of image processing, the contemplated medical imaging techniques, the applied classification approaches, and the types of medical images, among others. In this context, we present here a brief review of some of the relevant works.

In terms of image segmentation, in [27] authors propose a method applied to Computed Tomography (CT) chest images that are based on multi-agent deep reinforcement learning to sharpen the automatic masking process. The technique has been evaluated in a combined dataset collection, and compared against various similar-purpose state-of-the-art methods, all based on deep learning frameworks, achieving an accuracy of 97.1%. Other studies pursue the same objective of segmentation for COVID-19 CT images using deep learning architectures, like in [28] where authors use UNet++ as a feature extractor to achieve the purpose of segmentation, and later, combine it with ResNet-50 used as a backbone. In [29] authors evaluate the VGG-19 deep learning architecture applied to the segmentation of nodules in lung CT images. The proposed methodology shows promising results in a segmentation task that potentially shares relevant similarities with COVID-19, achieving a maximum of 97.83% accuracy with the SVM-RBF classifier.

A distinct approach involving COVID-19 CT image segmentation is presented in [30], where an extended segmentation-based fractal texture analysis composes the feature extraction phase united with other techniques, such as discrete wavelet transform. Then, an entropy-based genetic algorithm performs the feature selection, generating an optimal fused feature space, which is evaluated with various traditional classifiers. The method obtains the best accuracy with the naive Bayes classifier, presenting 92.6% of accuracy for a dataset obtained from radiopaedia. On the same dataset, the study in [31] employs two pre-trained deep learning models, AlexNet and VGG-16, for COVID-19 classification, not in a segmentation-based approach. Instead, they first apply a hybrid contrast enhancement technique and, later, fine-tune the deep methods. The features from both models are extracted and fused, and the optimal features are selected with an entropy-controlled firefly optimization method. Lastly, these features are, then, provided to various traditional machine learning classifiers, where the SVM achieved the best accuracy of 98%.

For chest X-ray images, [32] proposes a multi-class framework for detecting 15 types of diseases, including COVID-19. It implements a Convolutional Neural Network (CNN) for deep feature extraction, later these are fed, via transfer learning, to traditional machine learning classification methods for boosting the prediction results. When evaluating the technique, the authors combined two datasets, where the NIH Chest X-ray Dataset provides the samples for 14 types of chest-related diseases and the COVID-19 Chest X-ray Image Dataset provides the samples for COVID-19 X-rays. This two-way classification scheme saturates the learning accuracy on 87.8% with CNN and later improves to an accuracy of 99.8% with *k*-NN. In [33], authors combine three different sources of chest X-ray images with synthetic data from a Generative Adversarial Network (GAN) to obtain a balanced 15 disease classes (including COVID-19) dataset. Then, four independent deep learning models are evaluated concerning the classification capability, with ResNet-152 providing the higher accuracy of 87%. The work presented in [34] proposes a new Domain Extension Transfer Learning (DETL) approach to deal with the limitations presented by COVID-19 chest X-ray images available to that date. The DETL has been evaluated along with AlexNet, VGG-16, and ResNet-50 for a classification problem containing four classes. The overall best accuracy of 90.13% is obtained with VGG-16.

Other notable approaches to COVID-19 chest X-ray image classification should also be mentioned. In [35], authors address the problem by proposing a multi-scale BoDVW-based (Bag of Deep Visual Words) feature extraction, where the raw feature map is extracted from the 4*th* pooling layer of a VGG-16 pre-trained model, capturing detailed semantic relationships with three distinct kernels. The proposal is applied as a multi-class classifier and evaluated with four datasets in comparison to five other state-of-the-art methods. The results present a significant improvement in performance with a top accuracy of 90.29%. When considering improvements in explainability, the study in [36] exemplifies the ongoing research efforts to enhance the accuracy and interpretability of deep learning models for chest X-ray image analysis. By incorporating XAI (eXplainable Artificial Intelligence) techniques into a single lightweight CNN, researchers have provided a four-class classification model that outperformed the existing methods, presenting an accuracy of 95.94%, while providing medical radiology experts with noticeable tools for aiding in the interpretation.

## High-Level classification preliminaries

Here, the foundations for the Network-Based High-Level classification [10, 20] are presented with some of its properties and distinguishing features, which are essential to the proper conceptual understanding of the proposed method.

Generally, a data classification problem is presented in its supervised form, when there is a set of known labels (indicating the corresponding classes) for a subset of data. This set can be given to an algorithm to be used as the training set for the learning process; named supervised learning process in this scenario. With it, the algorithm can check the quality of the relations inferred between the attributes, and thus the learning is done by iteratively adjusting the parameters for those relations and minimizing the error. Ultimately, it achieves the best parameters, establishing a certain model for the data, with which it is possible to predict the proper class, mapping data samples to the expected class with a certain accuracy.

For the described inference process, the vast majority of data classification methods consider only the “physical features” from the provided data, e.g., distance, density, similarity, or distribution, to guide the learning process. Also, the act of learning can be understood as the establishment of some sort of decision boundary capable of segmenting the *n*-dimensional space of data into distinct regions that represent the expected classes. Therefore, in the context of a high-level classification, these methods are referred to as “low-level classification”.

Thereby, the central concept for the high-level classification contrasts with the prior methodology by focusing on the identification of intrinsic pattern formation from the provided data, instead of relying only upon physical relations between features. This conceptual change intends to allow the task of classification to be done by analyzing the data at an organizational and semantic level, no matter how similar or dissimilar when measured by the physical features of the data.

With this, it is expected that a high-level classification technique can explore non-trivial problems and scenarios, where the feature space of data is presented with complex geometric distributions (e.g., twisted shapes, overlapping, contexts, etc.). The process employed by low-level classification of determining regions and boundaries in the *n*-dimensional space on complex scenarios becomes an unnatural way of addressing and modeling these problems.

**Fig 1:**
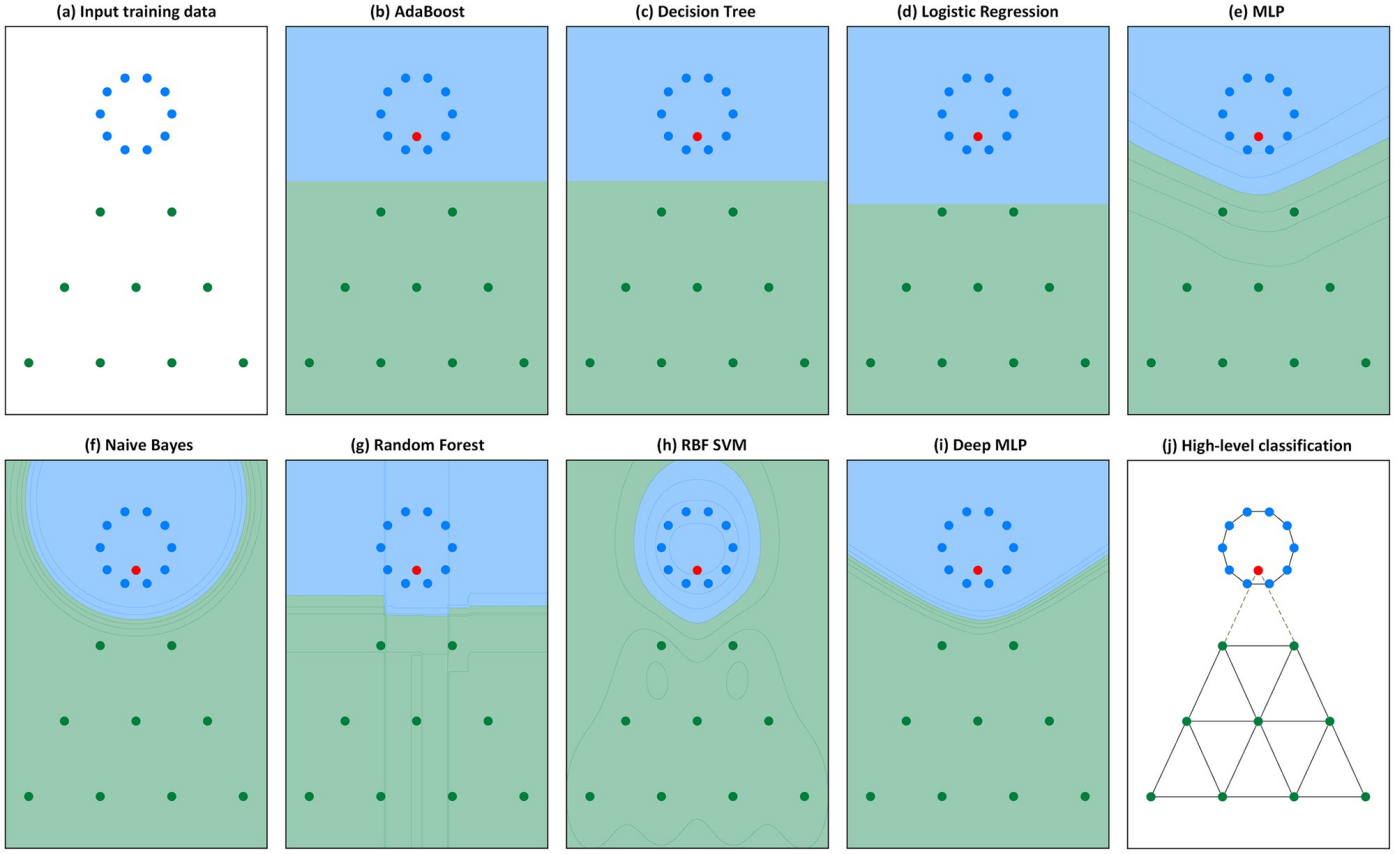
A simple non-trivial comparative classification scenario. The *red* sample is located in a dense *blue* region, despite presenting high conformity with the sparse *green* triangular pattern. (a) The input training data provided to all algorithms is presented in the figure. The red sample is provided only for the testing phase. (b-i) Various traditional machine learning techniques. All failed to predict the red testing sample into the green class. (j) The high-level classification technique is the only one to correctly assign the red testing sample to the green class.

In order to illustrate the salient features of the high-level pattern-based classification. We present the following simulation results on a toy dataset. Fig 1 illustrates an artificial and very simple scenario where the low-level classification techniques fail to infer the correct expected class for a certain data sample (in red). There are two classes: The blue data conforms to the circle pattern and the green data conforms to a triangular pattern. The training data, shown by Fig 1(a), is provided to various classical and state-of-the-art machine learning algorithms (Fig 1(b)–1(i)). After the training phase, it is possible to observe the varied decision boundary configuration strategies. However, when presenting the red testing instance, according to the traditional low-level learning mechanism of relying on purely physical relations, the testing sample is inferred as belonging to the blue circle class, delimited by its respective boundary. This occurs due to the high affinity of the testing sample and the blue class data elements according to the “physical” guiding measures: distance, density, similarity, and distribution.

However, when examining Fig 1(a), our mind intuitively perceives the red sample as belonging to the green triangular class in a much more natural way, as it exhibits a high degree of conformity with the pattern displayed by the green class. In accordance with it, Fig 1(j) shows that the proposed high-level classification technique correctly identifies the red testing instance into the proper green class. Since it constructs a distinct network for each of the classes, the testing sample is evaluated according to the conformity it presents to each class, being attributed to the one that causes less perturbation. In other words, the sample is attributed according to the pattern conformation to the class network. Thereby, the high-level classification technique gives higher relevance to pattern formation in the inference process, surpassing the guidance purely based on physical measurements.

The high-level classification approach proposed in [10, 20] addresses this concept by representing the input data as networks, where a distinct network is constructed for each one of the classes during the training phase. The prediction (testing phase) is performed with the insertion of the test sample, being evaluated, into each of the networks and, then, analyzing the conformity of the element with each network, concerning the caused perturbation in it. Thus, the test sample is assigned to that class where its insertion in the corresponding network causes the least variation of the measures under consideration.

In both works, a hybrid approach is implemented for the prediction phase, unifying the low-level classification, which can be implemented by any traditional classification technique, with the high-level approach, which explores the complex topological properties of the network built from the input data. The difference between these two works resides in the strategy to evaluate the network perturbance when inserting the testing element. In the work introduced in [10], the high-level classification is performed using three network measures: assortativity, clustering coefficient, and average degree. While in [20], the network perturbation is characterized by measures obtained from the dynamics of tourist walks, extracting the transient and the cycle lengths to describe network patterns.

Later, this concept is extended in [21, 22], where the low-level classification component has been eliminated and pure high-level classification techniques have been proposed. However, in those works, several network measures are employed in conjunction, introducing a large set of parameters representing the weight for each measure, which are hard to correctly determine. For this reason, we introduce a modified high-level classification technique using only one network measure, the communicability measure [23], which largely reduces the parameter calibration task while keeping great performance.

## Materials and methods

In this section, we present the proposed method for classifying chest X-ray images step by step. In brief, our method starts to apply feature extraction methods to both COVID-19 and normal X-ray images. This allows each image to be represented in the *n*-dimensional space by its feature vector. Then, the training phase is started by constructing a distinct network for each class (normal and COVID-19). Having those networks, a new unlabeled data sample can be evaluated in the testing phase. Given this sample, already, as a feature vector, it is incorporated into each network, and later, the level of caused perturbation is calculated with the communicability network measure. Lastly, the testing sample is associated with that class in which the sample caused the least variation in the measure.

**Fig 2:**
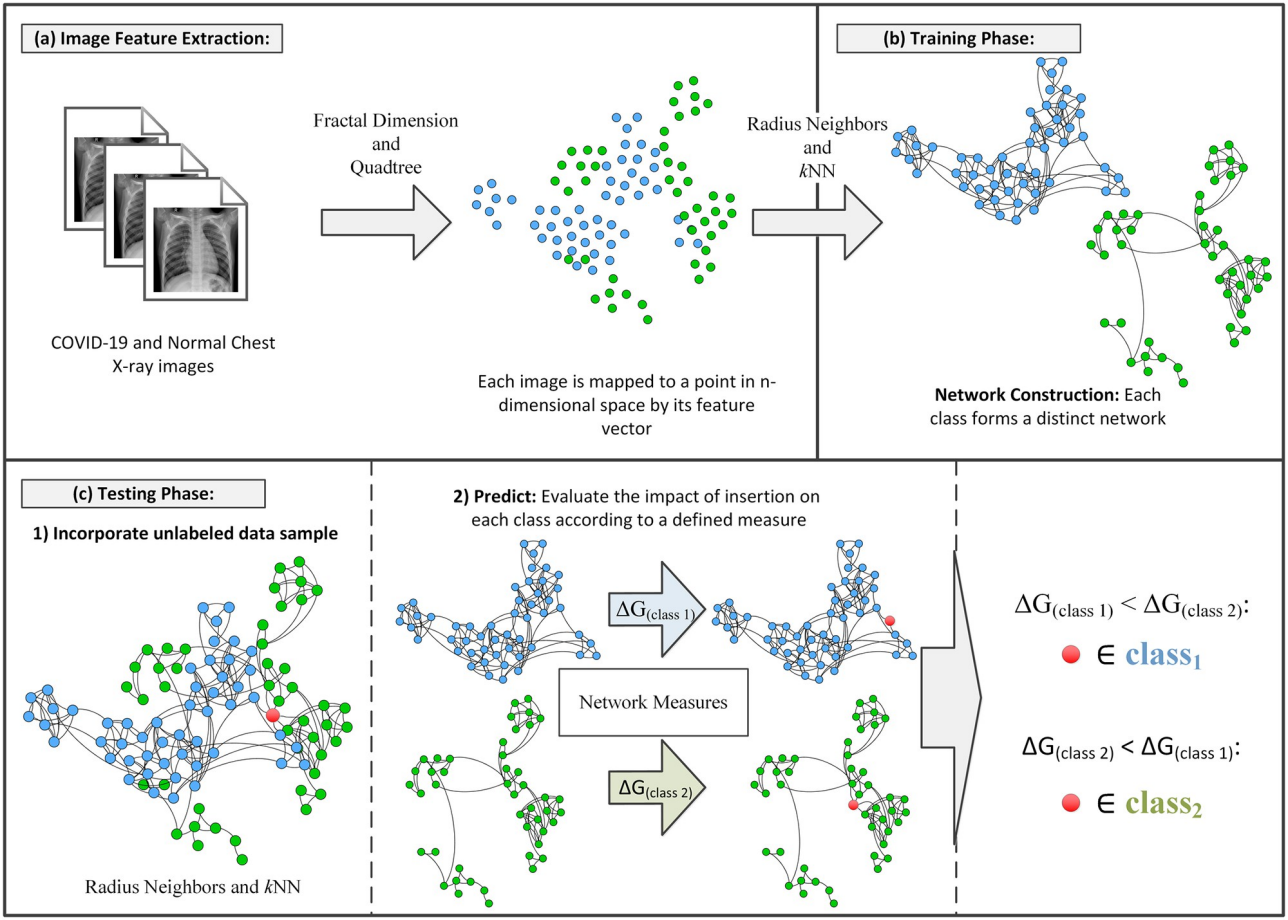
Overview of the modified high-level classification technique. (a) Image feature extraction phase maps images to points in *n*-dimensional space. (b) The training phase constructs the network for each of the classes; normal and COVID-19. (c) The testing phase incorporates the unlabeled sample to be evaluated by its impact on a defined network measure, predicting its membership to the network with the least variation of the measures under consideration.

An overview of the method is illustrated in Fig 2. The text that follows will cover all the phases in the figure, together with the foundations, motivations, and techniques that compose our method.

### Image feature extraction

When dealing with image processing, feature extraction is a common step, if not mandatory. Since each image contains a huge amount of information, it is important to find metrics or techniques to extract relevant characteristics of this image. Otherwise, dealing directly with the image pixels would be a hard task to treat variations of images in the same class and pose the classification problem in a high dimensional space that would drastically hamper the classification accuracy due to the curse of dimensionality and high computational complexity.

Usually, it requires some feature engineering to find appropriate or optimal features according to the specificities of the problem. We address this feature extraction process by analyzing two metrics that present promising characteristics to the applied problem, they are fractal dimension [17, 18] and quadtree [19].

#### Fractal dimension

Fractal dimension [17, 18] can be used to describe the geometrical complexity of the images. It allows the quantification of an image according to complexity analysis, where patterns of visual information contained in an image can be abstracted as fractal geometry, according to how the detail in a pattern changes with scale. Thus, the fractal dimension provides a measure that acts as an index of complexity, where a larger value indicates a higher complexity level, and, on the contrary, smaller values indicate lower complexity. These characteristics motivated the use of this measure as a feature extraction method in our work.

**Fig 3:**
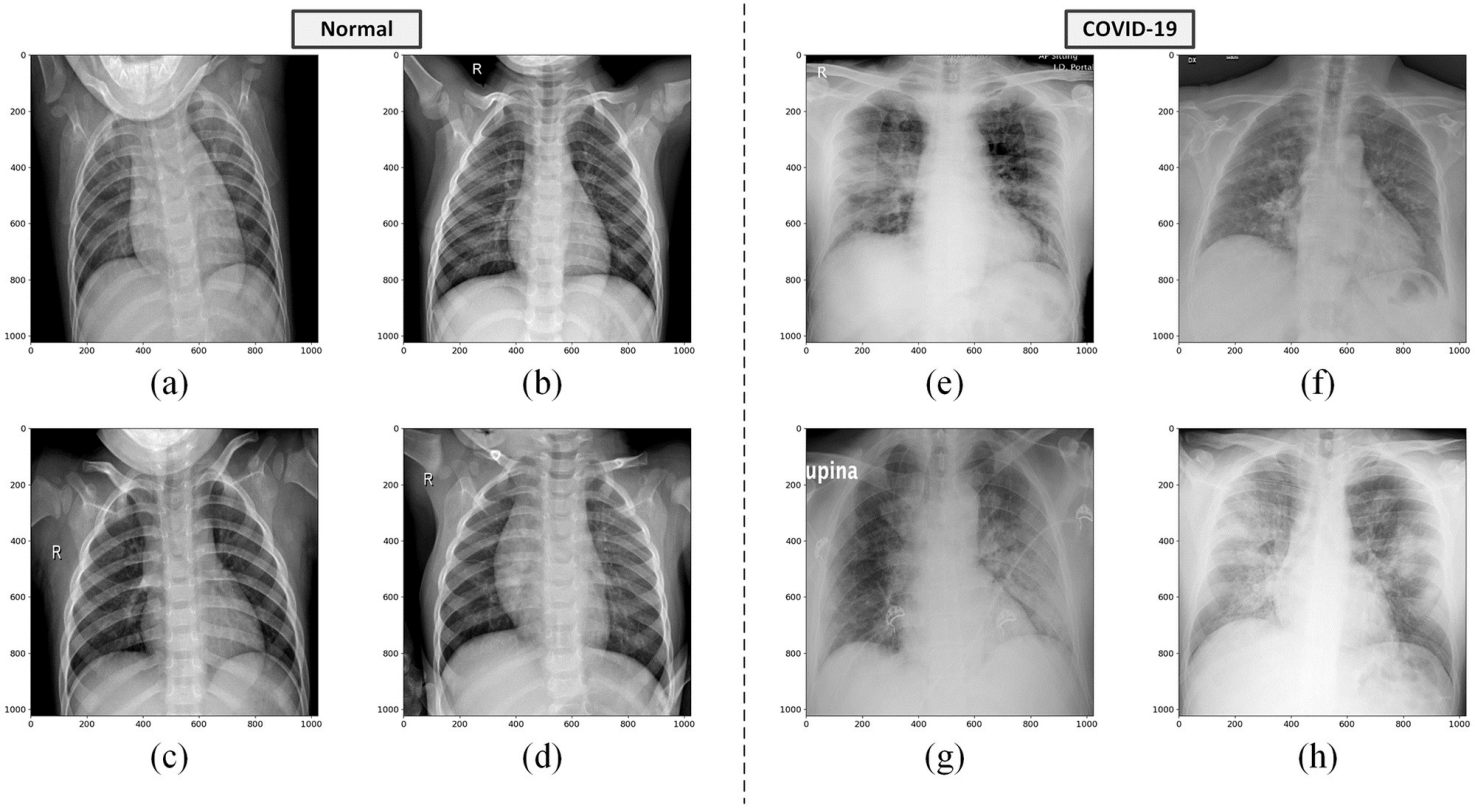
Chest X-ray images of healthy and infected COVID-19 lungs. (a-d) Healthy chest X-ray images. (e-h) COVID-19 chest X-ray images. The images are taken from the COVID-19 Radiography Database [37].

**Fig 4:**
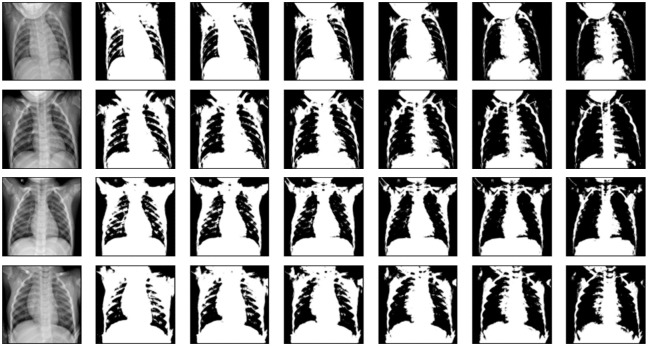
Four normal X-ray images and their corresponding binary images. The original gray-level images are leftmost. Each binary image, from left to right, is generated with threshold values 100, 110, 120, 130, 140, and 150, respectively.

**Fig 5:**
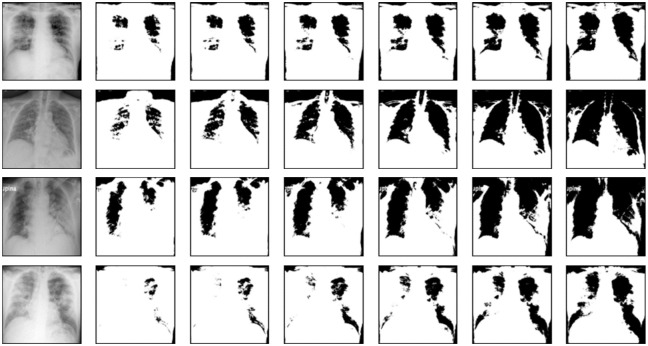
Four COVID-19 X-ray images and their corresponding binary images. The original gray-level images are leftmost. Each binary image, from left to right, is generated with threshold values 100, 110, 120, 130, 140, and 150, respectively.

For calculating the fractal dimension, we use the box-counting method [18], since it is widely used in fractal analysis. The method is applied to binary images. Therefore, given a gray-level chest X-ray image, we first generate several binary images simply using a series of increasing threshold values. Some examples of the original images are shown in Fig 3 and their corresponding resulting binary images can be seen in Figs 4 and 5.

Then, for each binary image, we cover the image with a grid and then count how many boxes of the grid are covering the pattern in the image. Then we repeat the process but use smaller boxes. By shrinking the size of the boxes repeatedly, we can accurately capture the structure of the pattern. The fractal dimension *D* is the slope of the line when we plot the value of *log*(*N*) against the value of *log*(*r*):

$$\begin{array}{c} D=\frac{log(N)}{log(r)} \end{array}$$ (1)

where *N* is the number of boxes that cover the pattern and *r* is the inverse of the box size.

Finally, for each gray-level image, we get a vector of values, each of which is the box-counting dimension of one of its binary images. Thereby, each vector acts as a feature vector that maps an image to a point in the *n*-dimensional space, as represented in Fig 2(a).

#### Quadtree

Analogous to fractal dimension, quadtree [19] can also be used to describe the geometrical complexity of the images. From a slightly different perspective, it allows the decomposition of the two-dimensional space, by partitioning it recursively into four new quadrants when a non-homogeneous area is found. This decomposition strategy leads to a few blocks of big size covering the vast homogeneous regions and many blocks of small size wrapping the very heterogeneous regions, richer in detail.

Then, the resulting distribution of block sizes and their corresponding quantity (occurrence) can be used to describe and compare images according to how homogeneous or heterogeneous they are. This is sufficient motivation for evaluating this algorithm as a complementary feature extraction method in our work.

Here, we apply the quadtree algorithm directly for each gray-level image and later get the histogram for the distribution of block sizes and quantities that compose the values for the feature vector. Again, each vector can map an image to a point in the *n*-dimensional space; Fig 2(a). Both feature extraction strategies can be employed separately or in conjunction, united in a mixture feature vector.

### The modified high-level classification technique

As the original high-level classification proposed in [10, 20], the modified high-level classification technique, introduced in this work, is also divided into two phases: the training phase and the testing phase. However, the modifications we propose reduce the complications of parameter calibration since the number of parameters is diminished. We implement two modifications in this respect. The first modification incorporates the strategy used in [22], where the two parameters (*k* and *r*) required for the network construction phase are reduced to one, with the radius (*r*) being determined according to the parameter *k*. The second improvement reduces the parameters in the testing phase with the employment of the communicability measure [23], which shows more robust performance when analyzing network perturbation for this specific applied problem.

In the training phase, we construct a distinct network for each class of image features, where the feature vector of each image is a node and the connections between nodes are formed by a technique that uses either *k*-Nearest Neighbors (*k*-NN) or Radius Neighbors (*RN*). Where the *k*-NN is used for sparse regions and *RN* for dense regions. An illustration of this phase is in Fig 2(b).

If a feature vector of an image has a small number of similar feature vectors of other images, the corresponding node in the network falls in a sparse region. In this case, this node is connected to its *k* most similar nodes. On the other hand, if a feature vector has a large number of similar ones, it falls in a dense region and is connected to all the nodes within a predefined similarity radius. Thus, the network construction criterion is defined by the following equation:

$$\begin{array}{c} N\left(x_{i}\right)=\{\begin{array}{c} RN\left(x_{i},Y_{x_{i}}\right),if\left|RN\left(x_{i},Y_{x_{i}}\right)\right|>k\\ kNN(x_{i},Y_{x_{i}}),otherwise \end{array} \end{array}$$ (2)

where *x*_*i*_ is a data sample (already mapped as a feature vector) and $Y_{x_{i}}$ denotes the class label of *x*_*i*_, indicating that only neighbors of the same class should be considered.

In addition, we define the *r* radius of Radius Neighbors by the following equation:

$$\begin{array}{c} r=median\{kNN_{dist}\left(x_{i},Y_{x_{i}}\right)\} \end{array}$$ (3)

where *kNN*_*dist*_ brings the distances from all *x*_*i*_ that are members of the same class in the training set to its *k*-NNs also in the same class.

Thus, the parameter *r* is calculated according to the value of *k*, being the median of the distance values returned when *k*-NN is applied to all points as the origin. This leaves the new proposed model with basically a single parameter, it is worth remembering that *k* is a natural integer parameter.

After the network construction, the testing phase can be initiated. The purpose of this phase is to predict to which class a certain unlabeled testing data sample should belong. The steps involved in this phase are shown in Fig 2(c). In this phase, we classify the unlabeled data samples one by one. Firstly, a new data sample is inserted (temporarily) into each of the two networks constructed so far based on the same principle used during the network construction phase, as represented by Eq 2.

Then, the same measure of each network after the insertion, *G*_*after*_(*class*_*i*_), *i* = 1, 2 is calculated and compared to the measure of each network (each class) before the insertion, *G*_*before*_(*class*_*i*_), *i* = 1, 2. Now, we have the impact of the insertion of the new sample to each class, given by,

$$\begin{array}{c} \Delta G\left(class_{i}\right)=\left|\right|G_{before}\left(class_{i}\right)-G_{after}\left(class_{i}\right)\left|\right|\;,\;i=1,2. \end{array}$$ (4)

Finally, the new sample is classified to class *j*, where

$$\begin{array}{c} \Delta G\left(class_{j}\right)=min\left\{\Delta G\left(class_{i}\right)\right\}\;,\;i=1,2. \end{array}$$ (5)

In our method, we use the average communicability measure $\langle M_{v_{i}}\rangle$ [23] as *G*_*before*,*after*_(*class*_*i*_), which accounts not only for the shortest paths connecting two nodes but also the longer paths with a lower contribution. The communicability $M_{v_{i}}$ from node *v*_*i*_ to all other nodes of the network is described by,

$$\begin{array}{c} M_{v_{i}}=\frac{1}{\left(N-1\right)}\sum_{j\in N}(\frac{1}{s!}P_{v_{i}v_{j}}+\sum_{k>s}\frac{1}{k!}W_{v_{i}v_{j}}),\;i\neq j \end{array}$$ (6)

where *s* is the length of the shortest path between *v*_*i*_ and *v*_*j*_, $P_{v_{i}v_{j}}$ is the number of shortest paths between *v*_*i*_ and *v*_*j*_ and $W_{v_{i}v_{j}}$ is the number of paths connecting *v*_*i*_ and *v*_*j*_ of size *k* > *s*. The reasoning behind this choice is that the shortest paths are significantly affected by structural changes in a network.

In other words, the new sample conforms to the pattern formed by network *j* if it doesn’t generate a larger perturbation to the network *j*. Observe that the new sample can even stay far from the elements of class *j* in the physical space.

### Algorithms

Here, we provide the algorithms for the modified high-level classification technique. Algorithm 1 describes the steps for the training phase, while Algorithm 2 refers to the testing phase.

This experimental setup is common to all supervised learning algorithms. In the training phase, the algorithm constructs a model from the provided labeled training data, and in the testing phase, it predicts the label for the queried unlabeled samples. Traditional machine learning approaches determine the classification model relying on physical characteristics of the data space, by the demarcation of decision boundaries.

Therefore, the main distinction of our approach resides in the fact that the classification model is now represented by a network formation process, resulting in a distinct network representation for each of the classes.

**Algorithm 1** Training phase: Modified high-level classification

**Input**: A given training dataset composed of *n* images and the corresponding label vector.

**Output**: A distinct network representing each of the classes.

1: Calculate the feature vector for each of the *n* images, using fractal dimension and quadtree

2: **for each** *class*_*c*_ **do**

3:  Calculate the *k*-NN data structure for all samples of *class*_*c*_

4:  $r\leftarrow median\{kNN_{dist}\left(x_{i},Y_{x_{i}}\right)\}$

5:  *G*(*class*_*c*_) ← Add an unconnected node representing each data sample of *class*_*c*_

6:  **for** *x*_*i*_ ∈ *class*_*c*_ **do**

7:   **if** $|RN(x_{i},Y_{x_{i}})|>k$ **then**

8:    *G*(*class*_*c*_) ← Connect *x*_*i*_ representative node with $\left\{RN\left(x_{i},Y_{x_{i}}\right)\right\}$

9:   **else**

10:    *G*(*class*_*c*_) ← Connect *x*_*i*_ representative node with $\left\{kNN\left(x_{i},Y_{x_{i}}\right)\right\}$

11:   **end if**

12:  **end for**

13:  $G_{before}\left(class_{c}\right)\leftarrow \langle M_{v_{i}}\rangle$

14: **end for**

In Algorithm 1, the “for” in Line 2 controls the construction of a network for each training class. In Lines 6 to 12, a data sample *x*_*i*_ is really inserted in the corresponding network according to the rules defined by Eqs 2 and 3. Line 13 calculates the average communicability of each network using Eq 6.

**Algorithm 2** Testing phase: Modified high-level classification

**Input**: A testing instance *x*_*t*_.

**Output**: The predicted label of the testing instance: ${\hat{Y}}_{x_{t}}$.

1: Calculate the feature vector for the testing instance, using fractal dimension and quadtree

2: **for each** *class*_*c*_ **do**

3:  **if** |*RN*(*x*_*t*_, *class*_*c*_)| > *k* **then**

4:   *G*(*class*_*c*_) ← Connect *x*_*t*_ representative node with {*RN*(*x*_*t*_, *class*_*c*_)}

5:  **else**

6:   *G*(*class*_*c*_) ← Connect *x*_*t*_ representative node with {*kNN*(*x*_*t*_, *class*_*c*_)}

7:  **end if**

8:  $G_{after}\left(class_{c}\right)\leftarrow \langle M_{v_{i}}\rangle$

9:  Δ*G*(*class*_*c*_) ← ||*G*_*before*_(*class*_*c*_) − *G*_*after*_(*class*_*c*_)||

10:  ${\hat{Y}}_{x_{t}}\leftarrow min\left\{\Delta G\left(class_{c}\right)\right\}$

11:  *G*(*class*_*c*_) ← Remove *x*_*t*_ representative node

12: **end for**

In Algorithm 2, Line 2 controls the insertion of a testing data sample *x*_*t*_ in each class network. Specifically, *x*_*t*_ is inserted into the network *class*_*c*_ by Lines 3 to 7, according to Eq 2. Line 8 calculates the average communicability measure after the insertion of *x*_*t*_. Line 9 checks the variation of the average communicability measure before and after the insertion, according to Eq 4. Thus, Line 10 determines the class to which *x*_*t*_ belongs based on the pattern conformation criteria. Lastly, Line 11 discards the testing sample by removing the corresponding node from both networks.

The computational complexity of the modified high-level classification technique can be determined as follows. We first examine the computational cost for the training phase, where a network is constructed to represent each class of the provided training dataset. This phase initially requires a distance matrix for the *k*-NN and Radius Neighbors, presenting a complexity of *O*(*n*^2^) to be constructed, and *O*(1) to query the neighbors of a data sample. Next, the median to determine the radius *r* is calculated in *O*(*n*). Thus, the network formation can be concluded with *O*(*n*^2^), and the network measure can be calculated with *O*(*M*). Resulting in a total computational complexity of *O*(*n*^2^ + *M*) for the entire training phase.

Concerning the testing phase, given that the class networks are already formed, the testing instance requires only one query to the *k*−NN and Radius Neighbors distance matrix to find its neighbors and connect to them, which can be done in *O*(1). Thus, the incorporation of the testing sample into the networks requires only *O*(1), and, finally, a new calculation of the network measure is required. Thereby, the entire testing phase demands a total computational complexity of *O*(*M*).

In this paper, the communicability measure is applied. It consists of calculating the *l*-th power of the adjacent matrix and, then, the eigenvalues of each class, where *l* is the shortest distance between two nodes. Generally, *l* ≪ *n*, therefore, the complexity order of $O\left(M\right)=O({\left(max\left(n_{C_{i}}\right)\right)}^{3})$, where $max(n_{C_{i}})$ is the number of data samples of the largest class. Certainly, $max(n_{C_{i}})<n$. Obviously, the computational complexity can be reduced if we apply other network measures with lower complexity orders.

## Results

In this section, we present the computational results of chest X-ray image classification using the proposed method. Initially, we define the database used in the simulations, and its composition is characterized in detail. Next, the two complexity measures (fractal dimension and quadtree) are analyzed to check their capabilities as feature extractors, and, then, the mixture of these feature extractors is evaluated. Later, a network measure analysis is conducted to verify which one presents the greater potential to distinguish the networks. And, lastly, the classification results are exemplified and compared to other state-of-the-art techniques.

### Database

All radiographic images used in this paper have been obtained from a public data source: the COVID-19 Radiography Database, Version 1 [37–39]. The entire database contains 219 COVID-19 positive images and 1341 normal chest X-ray images. Each of the images contains 1024×1024 pixels with 8 bits of depth gray-scales.

For the simulations of this paper, we construct a balanced dataset by randomly selecting 150 healthy lung images and 150 COVID-19 images. Therefore, this dataset contains a total of 300 samples. During the classification experiments, the dataset is randomly split in the training and testing set, with a ratio of 9: 1; resulting in 270 samples for the training, and 30 for testing. For each algorithm under comparison, the classification results are averaged over 50 executions. We denote the two classes of this dataset as “normal” and “COVID-19”. Fig 3 shows some examples of the normal and COVID-19 chest X-ray images, respectively.

### Analysis of fractal dimension as a feature extractor

From Fig 3, we can observe that the healthy lungs X-ray images present a clearly distinct pattern when compared to the COVID-19 images. The COVID-19 lungs present the formation of filaments that spread through the entire X-ray image as an opacity texture that impairs the proper visualization of anatomical details. Therefore, since the fractal dimension is a geometrical complexity measure we expect that it will capture this disparity in visual information patterns. Thus, we extract the fractal dimension to compose feature vectors for characterizing the two classes, where larger fractal dimension values are expected for those patterns that indicate a higher complexity level.

In order to calculate the fractal dimension (box-counting dimension in this paper) for a gray-level image, first, we generate a series of binary images from it imposing a series of increasing threshold values, and then we calculate the box-counting dimension for each binary image. These thresholds range from 100 until 150, with increments of 10, being a total of 6 thresholds, and consequently generating a feature vector with 6 fractal dimension values for each image. Figs 4 and 5 show the binary images of the normal and COVID-19 gray-level images, respectively, corresponding to those original images from Fig 3. The binarization also shows the loss of the anatomical details for the COVID-19 lung X-ray images in comparison to normal images, especially for lower threshold values.

**Fig 6:**
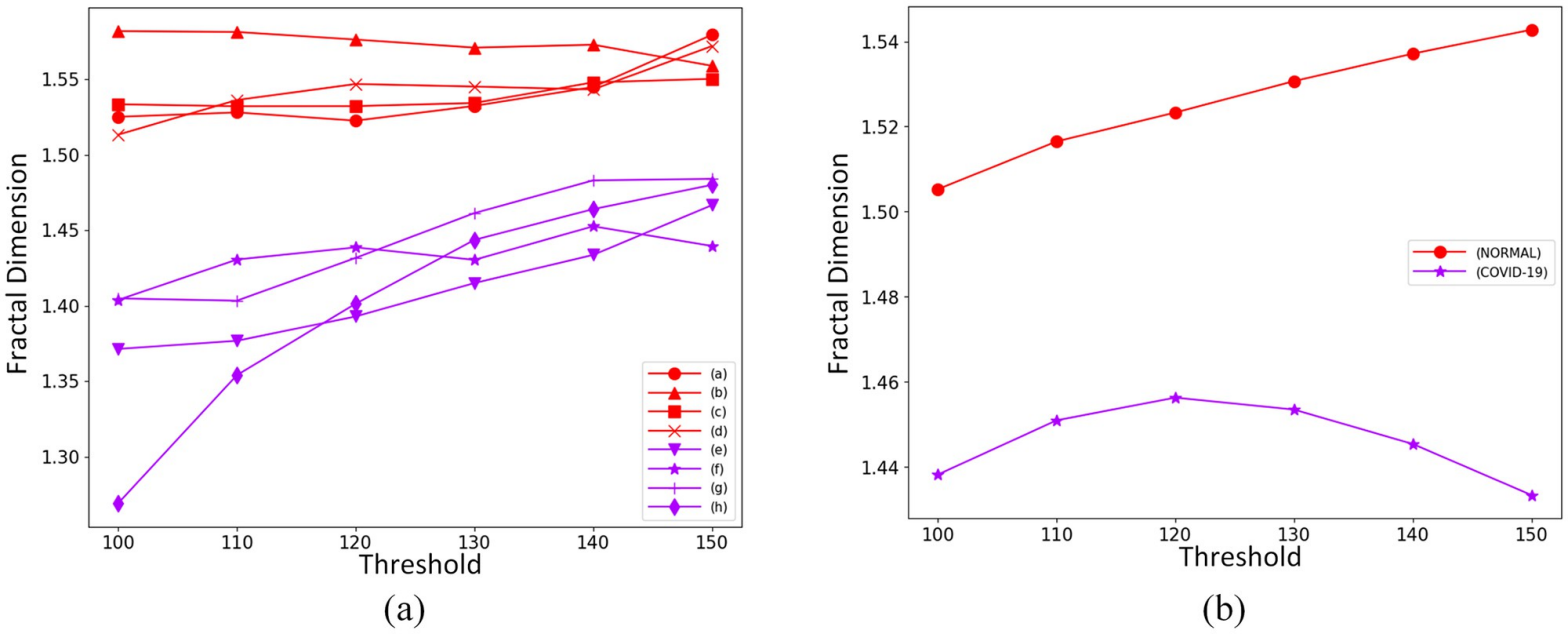
Calculated fractal dimension values. (a) Fractal dimension values of binary images for the 8 gray-level images shown in Fig 3, where *red* curves represent normal, and the *purple* represent COVID-19. (b) Mean value of the fractal dimensions for all images of the database.

A box-counting dimension curve can be generated for each original gray-level image to illustrate the behavior of fractal dimension values. Fig 6(a) shows the calculated box-counting dimension curves for the 8 original gray-level images from Fig 3, while Fig 6(b) plots the mean value for fractal dimensions of each class of images, considering the calculated measures for all the 150 images of each class. From these figures, we see that the normal images and the COVID-19 images have quite different complexity levels in terms of fractal dimension. Thus, as we expected, the fractal dimension measure acts as a good feature extractor differentiating the images concerning those distinct exhibited visual patterns.

### Analysis of quadtree as a feature extractor

In a complementary analysis to the fractal dimension perspective, we can observe that the normal and COVID-19 lung X-ray images, illustrated in Fig 3, also show a very distinct spatial distribution of details. By a simple visual inspection, it is possible to notice that the opacity texture present in COVID-19 images seems to cause a significant reduction in the heterogeneity of the lung X-ray image when compared to the normal images that are much richer in detail. Then, the quadtree algorithm can also be applied as a feature extractor to quantify these homogeneity differences, based on the values obtained from its histogram for the distribution of block sizes and quantities determined by its partitioning.

To obtain the histogram of values for the block distribution, the quadtree algorithm is directly applied to the gray-level image, with no need for binarization. Then, the histogram of block distribution is analyzed for block sizes of 1 until 64, with increments of 2. The 7 resulting values compose the feature vector for each inspected image.

**Fig 7:**
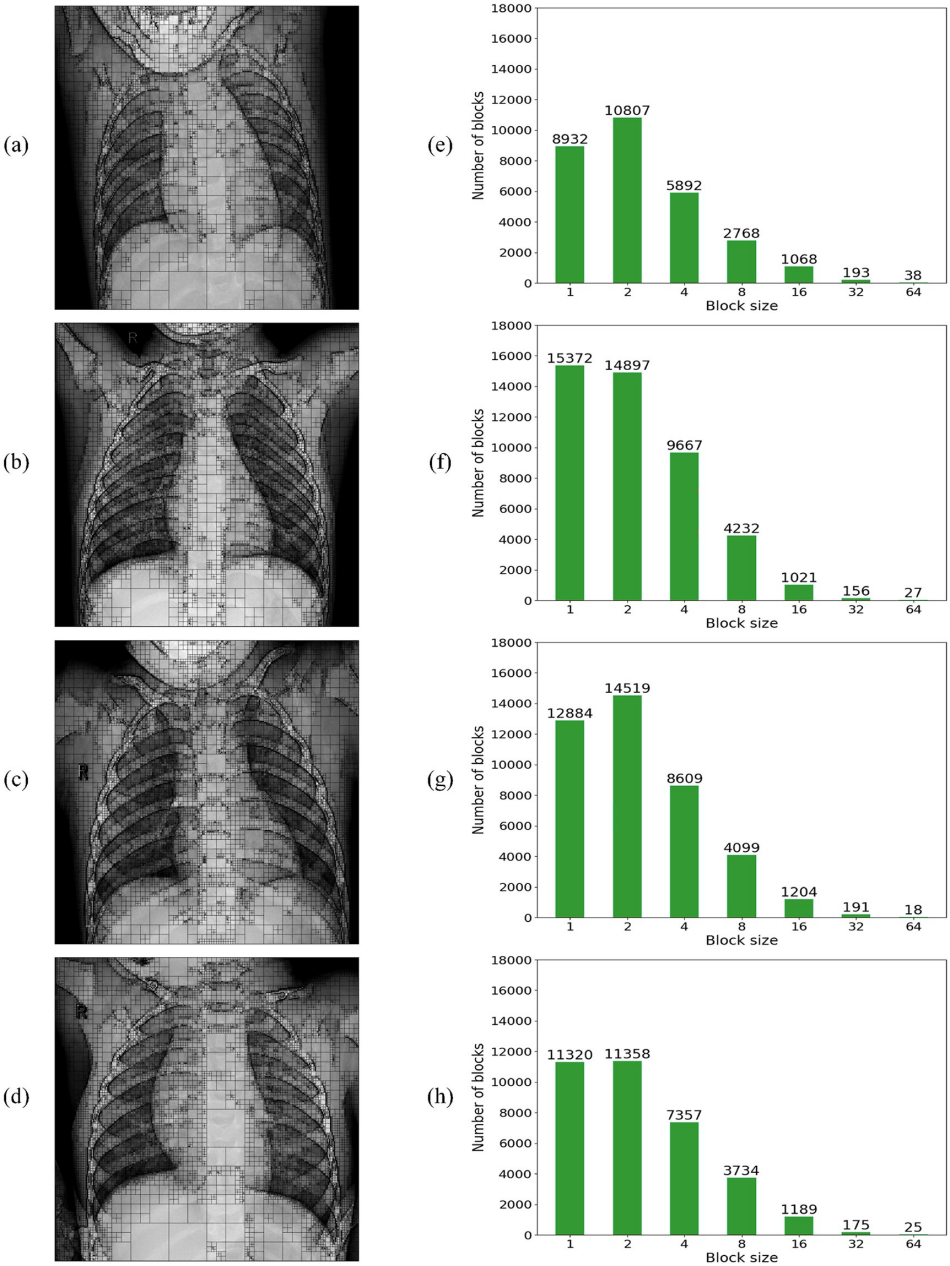
Quadtree analysis of four normal X-ray images. (a-d) Quadtree division of the 4 normal X-ray images shown in Fig 3. (e-h) Quadtree block size distribution corresponds to each of the images on the left.

**Fig 8:**
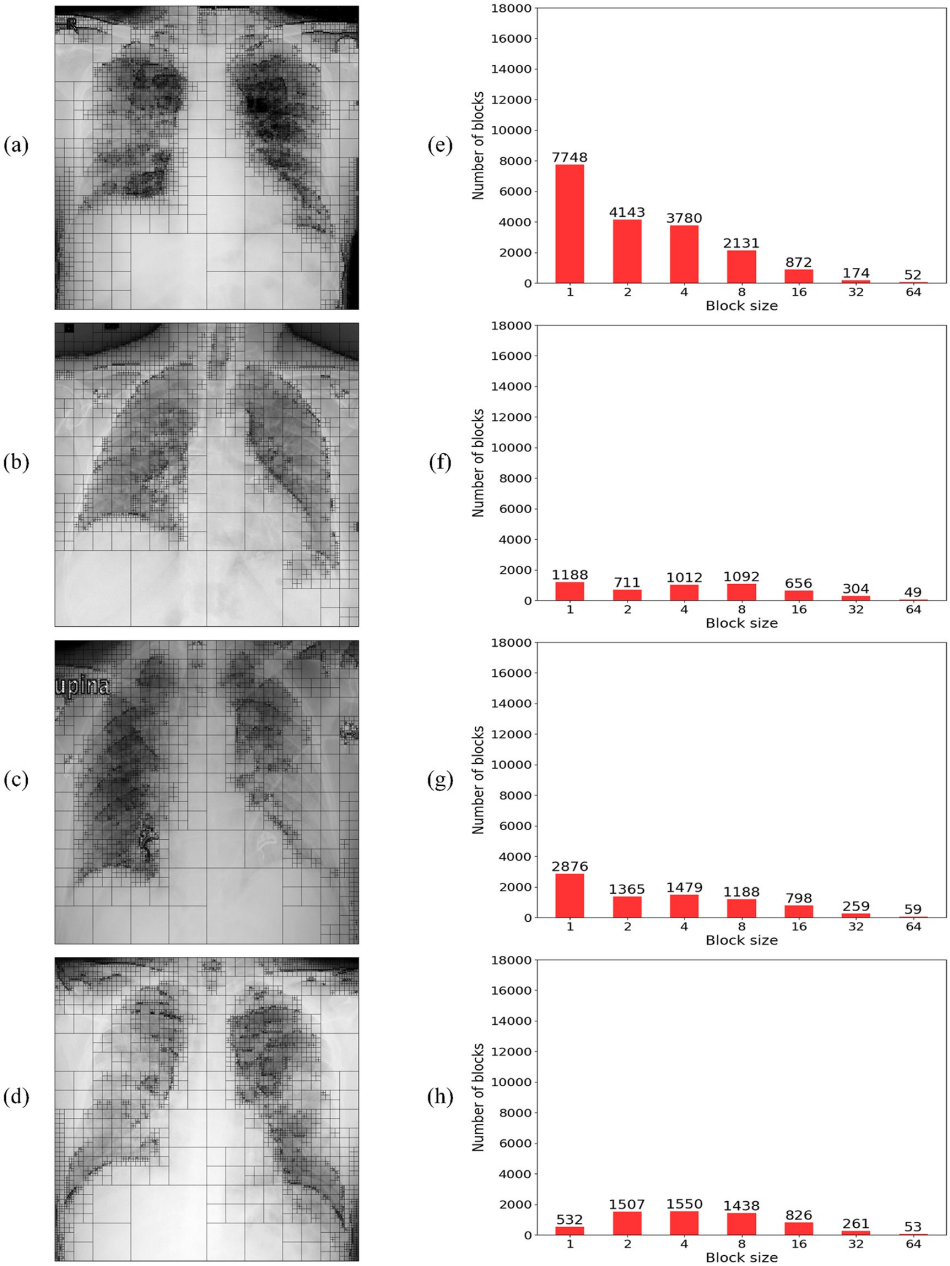
Quadtree analysis of four COVID-19 X-ray images. (a-d) Quadtree division of the 4 COVID-19 X-ray images shown in Fig 3. (e-h) Quadtree block size distribution corresponds to each of the images on the left.

The results for the quadtree division and the corresponding histogram of block distributions obtained from the same set of original images from Fig 3 are illustrated in Figs 7 and 8 for the normal and COVID-19 class samples, respectively. From both figures, we can observe that the lung X-ray images for normal class samples, Fig 7, always exhibit a higher number of blocks with a small size and a lower number of blocks with a big size when compared to images from COVID-19 class, Fig 8, which behave oppositely. Since more big blocks result in fewer partitions, as a consequence, the COVID-19 images also present fewer partitions in total.

**Fig 9:**
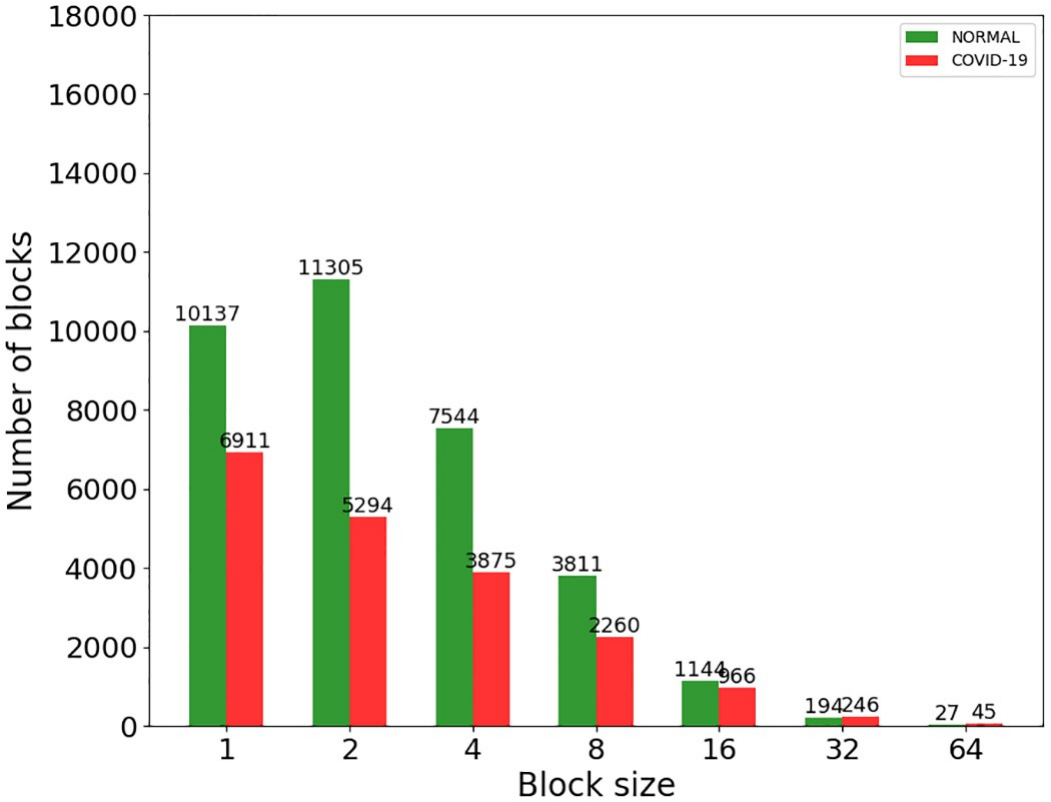
Histogram for the mean values of block sizes in quadtree division. The mean values are calculated for all images of the database. The *green* bar refers to the normal class images, while the *red* bar refers to COVID-19 X-ray images.

Fig 9 illustrates the average number of blocks, extracted by quadtree, according to the block size for all the 150 images of each class in the dataset. Where the green bar represents values for the normal class of images, and the red bar represents the values for the COVID-19 class. It can be seen that in the normal category images when the blocks are relatively small, the number of blocks is large, that is, the details are richer and the edges are clearer. On the contrary, in the COVID-19 category images, the smaller the blocks, the corresponding quantity is relatively small; being scarce the occurrence of small blocks, since the detail level is not as rich as in the normal category. Here, the feature vectors obtained from quadtree also show promising characteristics to differentiate images from both classes, normal and COVID-19.

### Mixture of feature extractors

Since both measures, fractal dimension, and quadtree, proved promising to characterize specificities for the elements of each class, we have analyzed those features according to the exhibited parameters for the spatial distribution of each specific class in its *n*-dimensional feature space. For a proper comparison, the measures are numerically normalized to the [0, 1] interval, and the separability in the feature space is characterized by the calculation of the mean value of the distances and the standard deviation of these distances for the elements of each class independently.

In addition, a mixture of those features is also evaluated by directly concatenating the feature vectors from both measures. Thus, the mixed feature vector is composed of those 6 values originally from fractal dimension for the various binarized images concatenated with the 7 values from the histogram of block distribution of the quadtree, for block sizes of 1 until 64, with increments of 2. Resulting in a mixed feature vector of 13 values for each image.

All the obtained values are presented in Table 1, where the evaluated feature vectors are rows and the mean values and standard deviation are at the columns, always with the pair of values by each corresponding class, normal and COVID-19, given respectively. From the table values, we can observe that fractal dimension and quadtree measures behave quite similarly in the capacity of separating the feature space, with a very similar absolute difference for mean distance and standard deviation for the class differentiation. Thus, from the mixed features perspective, the presented values evidence a more distinguishable absolute difference for the mean distance between classes, while maintaining a similar absolute difference for the standard deviation.

**Table 1:** Comparison of the feature extractors.

Feature	Mean distance		Standard deviation	
	Normal	COVID-19	Normal	COVID-19
Fractal dimension	0.193	0.431	0.105	0.230
Quadtree	0.351	0.569	0.171	0.277
Mixed features	0.414	0.733	0.170	0.315

Comparisons are performed in vector space to analyze a particular feature extractor, where the numerical values are calculated from the average of the distances among all vectors of a class, for all its images in the dataset.

These results support the use of the mixture of both measures as the input feature vector for our modified high-level classification technique, especially for the network construction in the training phase; Fig 2(b). As a result of the concatenation of features, the mixed feature vector has an increase in the number of dimensions, which does not incur problems related to the curse of dimensionality for our proposed technique since the feature vectors are only inputs for the network construction, i.e. these features are mapped to a complex network (graph).

### Network measures analysis

Revisiting the proposed high-level classification technique steps, after the training phase, the two classes, normal and COVID-19, are already represented by their own distinct network, having mapped the points in the feature space to their corresponding networks through the combination of Radius Neighbors and *k*-NN techniques; see Eq 2. And, as discussed in previous sections, the technique should, then, be able to perform the prediction of new elements through the steps illustrated in Fig 2(c). First, extract features of the input image, resulting in the mixed feature vector, and with it, incorporate the unlabeled data sample separately into both networks representing the classes; normal and COVID-19. With that, the prediction can be executed by analyzing the impact that the incorporation of the sample caused on a specified network measure before and after its insertion.

In this scenario, many network measures can potentially address this differentiation to support the evaluation task in the proposed framework, simply, any measure that is robust enough to capture the intrinsic network structure perturbance between the before and after incorporation of the unlabeled sample. From another perspective, the network measure aptitude can be evaluated according to its capability to directly distinguish the networks representing the normal and COVID-19 classes.

With this in mind, a set of network measures is calculated and analyzed for the dataset to compare which best distinguishes the network structure for the normal and COVID-19 networks. The obtained values can be observed in Table 2, where the rows represent the normal and COVID-19 classes and each column refers to a specific network measure. Thus, the calculated measures are Average Degree, Average Clustering Coefficient, Transitivity, Global Efficiency, and Communicability.

**Table 2:** Network measures analysis.

	Average Degree	Avg. Clustering Coefficient	Transitivity	Global Efficiency	Communicability
Normal	6.382	0.382	0.366	0.319	37.981
COVID-19	4.940	0.436	0.404	0.258	0.198

Various network measures are calculated to compare the networks constructed for each class of the problem; normal and COVID-19.

Specifically for the network measure analysis experiment, the normal and COVID-19 networks are constructed from all 300 data samples, representing the 150 available elements in each class of the dataset. To clarify, since the primary objective of the experiment is to assess the discriminative power of network measures within each representative network, there is no need for a training-test split configuration in the experimental design. The feature vectors provided to the network construction phase are obtained from the mixture of fractal dimension and quadtree for each original image. Additionally, all the resulting values in Table 2 are obtained for networks constructed with the parameter *k* = 5 of the *k*-NN technique, observing that the *r* parameter for Radius Neighbors is calculated from the Eq 3.

Observing Table 2 values, the communicability measure values stand out as those that easily distinguish both networks numerically, being other values with a much minor absolute numerical difference. This corroborates with the characteristics presented in [23] for the communicability measure, which gives evidence that it is capable of describing both the global and local network scales simultaneously. Furthermore, in [23], the communicability measure shows promising features for evaluating the structure-dynamic relationship of networks, which can have common properties to our tasks of evaluating the measure’s impact on the network before and after unlabeled sample incorporation.

### Classification results

**Fig 10:**
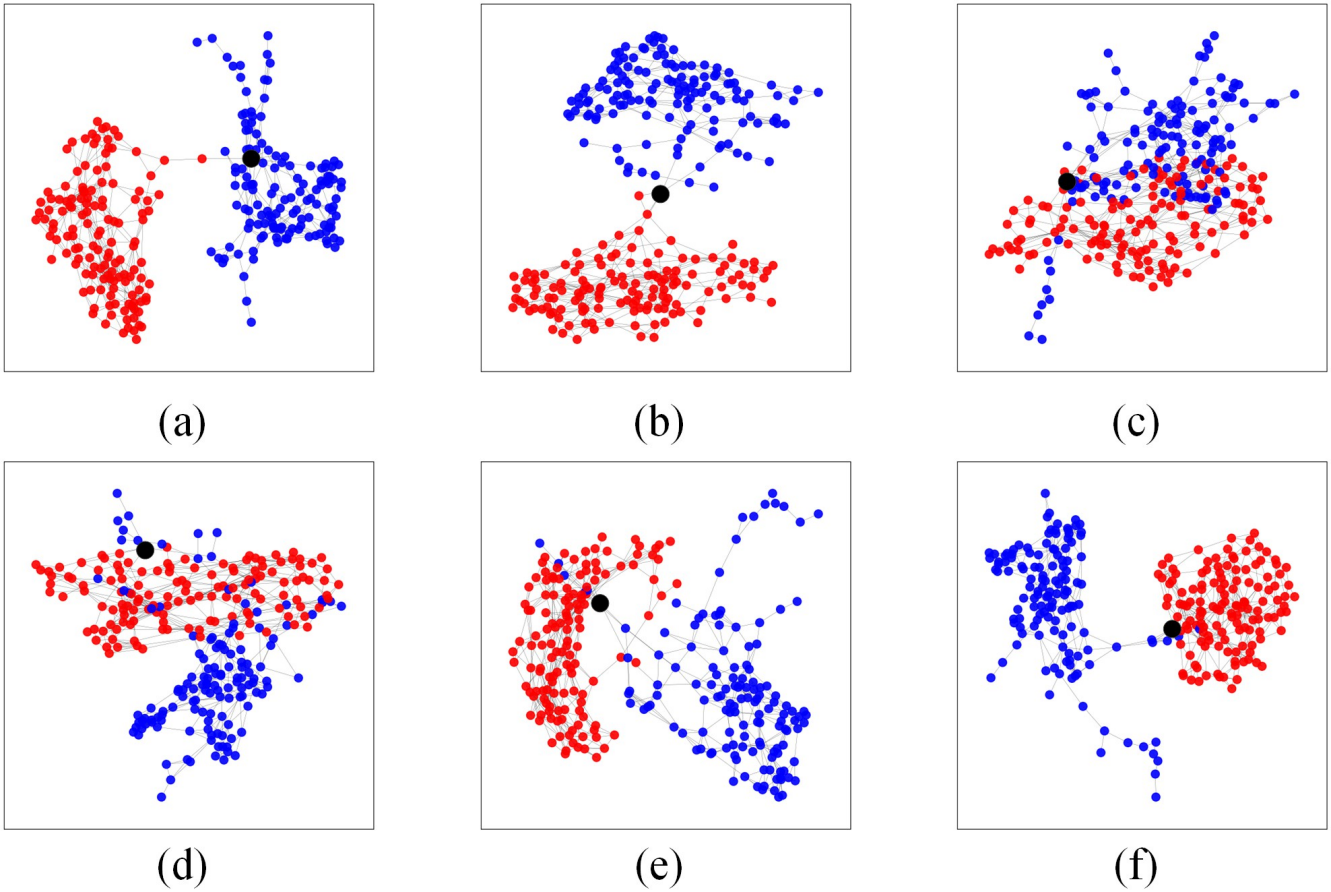
Six classification prediction examples. Six examples of classification prediction are randomly selected from the 30 testing dataset. In each of the six subfigures, the *red* network is formed from training samples of the normal class, and the *blue* network is formed from training samples of the COVID-19 class. For illustrating the classification process, in each of the subfigures, the *black* node is the testing node, which is briefly inserted into both networks in order to be evaluated.

Having constructed the networks to represent both classes, normal and COVID-19, in the training phase with a training-test configuration of ratio 9: 1, and defined the communicability measure as a suitable network measure to assess the impact of the incorporation of new samples, here, we first provide an illustrative demonstration of the prediction process by the proposed method for just 6 examples randomly picked from the testing dataset of 30 samples. These examples are illustrated in Fig 10. The red nodes represent the network for the normal class, the blue nodes represent COVID-19, and the black nodes are the testing samples. The parameters for the training phase are *k* = 5 for the *k*-NN technique and *r* = 0.1703 for Radius Neighbors, observing that this parameter is obtained directly from the Eq 3.

The prediction is performed through the analysis of the conformity of the testing data sample with each of the two networks, using the communicability measure [23] and comparing its values before and after the insertion of the sample. Thus, the new element is associated with that class it conforms better (or provokes less perturbation), which is represented by the lowest value of Δ*G*.

Table 3 reports the numerical results and the corresponding predicted class for all six examples in Fig 10. The communicability values for the generated networks are 44.8541 and 1.9568 for the normal and COVID-19 classes, respectively. Note that the communicability value prior to each sample evaluation is always the same since the classification occurs during the testing phase, where the networks representing each class are already constructed and the testing samples are not incorporated into the model (network) after each prediction.

**Table 3:** Evaluating the six classification prediction examples.

Sample	Normal			COVID-19			Predicted Class
	Before	After	Δ*G*(*class*_*normal*_)	Before	After	Δ*G*(*class*_*COVID*−19_)	
(a)	**44.8541**	44.1975	0.0148	**1.9568**	1.9698	**0.0065**	COVID-19
(b)		44.2862	**0.0126**		1.9308	0.0132	Normal
(c)		44.3288	0.0117		1.9484	**0.0042**	COVID-19
(d)		44.3399	0.0114		1.9498	**0.0035**	COVID-19
(e)		44.4804	**0.0083**		1.9316	0.0128	Normal
(f)		44.1979	0.0146		1.9413	**0.0079**	COVID-19

Evaluation of samples from Fig 10(a)–10(f) according to the communicability measure calculated before and after the training sample insertion, for each of the classes.

For the testing data sample shown in Fig 10(a), the impacts for the insertions are calculated as Δ*G*(*class*_*normal*_) = 0.0148 and Δ*G*(*class*_*COVID*−19_) = 0.0065, therefore, since this sample causes less impact on the COVID-19 network, it is classified as belonging to this class. Regarding the second example shown in Fig 10(b), Δ*G*(*class*_*normal*_) = 0.0126 and Δ*G*(*class*_*COVID*−19_) = 0.0132, then, the sample is classified into the normal class. For Fig 10(c), Δ*G*(*class*_*normal*_) = 0.0117 and Δ*G*(*class*_*COVID*−19_) = 0.0042, resulting in its prediction to the COVID-19 class. The Fig 10(d) refers to Δ*G*(*class*_*normal*_) = 0.0114 and Δ*G*(*class*_*COVID*−19_) = 0.0035, and the corresponding sample is also predicted to the COVID-19 class. For the sample in Fig 10(e), Δ*G*(*class*_*normal*_) = 0.0083 and Δ*G*(*class*_*COVID*−19_) = 0.0128, being classified into the normal class. Lastly, Fig 10(f) with Δ*G*(*class*_*normal*_) = 0.0146 and Δ*G*(*class*_*COVID*−19_) = 0.0079 has its sample associated with the COVID-19 class.

Next, we perform a series of simulations to measure the performance of our modified high-level classification technique in comparison with several state-of-the-art techniques. All the simulations are averaged over 50 executions with the training and testing set randomly split in a ratio of 9: 1 for the 150 normal and 150 COVID-19 X-ray images of the dataset. The obtained results are shown in Table 4.

**Table 4:** Classification accuracy comparison.

Classification Technique	Accuracy	F1-Score
AdaBoost	93.9%	0.917
Decision Tree	93.4%	0.908
Deep Learning (ResNet-50)	98.0%	0.972
Logistic Regression	96.8%	0.934
Multilayer Perceptron	89.1%	0.893
Naive Bayes	89.6%	0.907
Random Forest	93.7%	0.902
SVM	94.7%	0.926
**Modified High-Level Classification Technique**	97.0%	0.953

For all the experiments, each of the classes (150 normal and 150 COVID-19 images) is randomly split into two subgroups: the training group (135 images) and the testing group (15 images). The results are averaged over 50 executions with randomly selected training and testing samples each.

It is worth observing that the training phase of the proposed method is accomplished with the construction of one independent network to represent each of the classes. During this phase, the algorithm only has access to the data from the training samples. There is no interaction between training and testing sets in the network construction phase. In other words, there is no mixing between training and testing sets, and all the testing samples are unseen by the constructed classifier. Once the training phase is completed, the “base” network that represents a class never changes. During the testing phase, an unlabeled sample is temporarily incorporated into both networks just for the purpose of calculating the impact in the network measure (communicability measure). With that, the sample is predicted as pertaining to that network it causes less perturbation characterized by the least variation in the network measure or, in a complementary interpretation, to that network in which the sample best conforms to the patterns expressed in the network topology. Lastly, the algorithm discards the testing sample by removing the corresponding node from both networks before proceeding to the next sample, preventing data leakage at the model level.

As shown in Table 4, our algorithm presents a competitive performance in terms of classification accuracy compared to several traditional classification methods for COVID-19 identification. The proposed technique achieves an average accuracy of 97.0% and an average F1-Score of 0.953, which exceeds all other techniques, except for the Deep Learning (ResNet-50) [40] that achieved 98.0% and 0.972, respectively.

Undoubtedly, the last decade’s impressive breakthroughs in image classification tasks have been due to the evolution of deep learning architectures, thus the ResNet-50 superior result is totally comprehensible and expected. However, it requires massive computing power [41] and diverse computational strategies to optimize its training convergence, e.g., transfer learning and fine-tuning of the hyperparameters. On the contrary, our technique, although requires large memory space for a big network, shows a simpler setup, involving the tuning of only a single parameter *k*, resulting in a much more manageable and explainable technique, in which is possible to visualize the class structure represented directly as a network.

Compared to all other surpassed techniques, the greater average accuracy of our technique is due to the fact that it creates a distinct network representation for each class of the problem, which intrinsically captures patterns, and other organizational and semantic level information since it is a *high-level classification* technique [10]. In contrast, all these surpassed techniques are robust, well-known, and consolidated, but rely only on physical characteristics, such as similarity, distance, or distribution to define data classes.

## Conclusion

In this work, we present a new network-based high-level classification technique. From the simulation on the artificial dataset, we see clearly that the network approach can capture data patterns even if the data sample falls within another class measured by the physical feature (the similarity or distance feature in this case). On the other hand, classical and state-of-the-art classification techniques cannot recover this data sample from another class. This is because those techniques only consider the physical features of the input data. Such a salient feature implies that the proposed network approach may provide an elegant solution for invariant pattern recognition problems, such as face recognition, where large variations among data samples appear. The simulations on the COVID-19 image dataset show that the proposed network-based technique achieves a similar level of classification precision as the deep learning technique. However, deep learning techniques, in general, do not have an explicit explanation of the classification decisions. On the other hand, the proposed network-based technique gives a straightforward reason why a data sample is classified into a determined class. Specifically, the proposed technique classifies a data sample by checking whether it conforms to the pattern formation of each class. Here, a network is constructed for the training samples of each class, and the pattern of each class is characterized by network measure(s). The extraction of patterns from X-ray images through complex network construction and the subsequent analysis of the impact on network measures when a new testing sample is briefly incorporated shows that pattern identification is an efficient and robust way for class prediction. This leads to new possibilities not only for classification tasks but also empowers new perspectives for the analysis of the structure of the data related to an applied problem.

Although the proposed technique also employs the concept of “network” as a key data structure to perform the classification, there are considerable distinctions in the purpose of these network representations when compared to deep learning architectures. As a fundamental difference, deep learning architectures usually employ networks with fixed topology (fixed number of nodes, fixed layers, all-to-all connections, etc.), while our technique constructs its networks according to the presented training data, resulting in a complex network without restriction on the topology. Therefore, our technique constructs an independent network to store a representation for each of the classes, since it is expected that each class presents inherent patterns and relationships. Furthermore, given that deep learning architectures use fixed networks as an information processing paradigm, at least part (some layers) of these “deep” networks require the adjustment of a large number of weights during the training phase. These weight adjustments represent changes in edge values connecting the “nodes” and, ultimately, will represent the learning and adaptation to the presented data. In contrast, the networks constructed by the modified high-level classification technique directly represent the data of a certain class, and the data patterns are characterized by complex network measures.

Furthermore, even though the proposed technique presents salient features in semantic data classification, it requires large memory space to store the large datasets and the corresponding constructed networks for each class. It may also take a long time to calculate network measures to characterize data patterns on large-scale networks. In future works, a new approach to pattern identification will be tested to enable local sensing for the references in the vicinity of the testing node instead of the entire network, to significantly reduce the computational cost with the network growth. In addition, the technique will be extended to include algorithms for coarsening and uncoarsening phases [42, 43] for the constructed networks to deal with large-scale problems. Therefore, when addressing the original problem with multiscale analysis, it’s expected to better control computational costs while maintaining a compatible accuracy. Additionally, we will also investigate the possibility of subdividing each data class into more than one network, enabling the identification of subpatterns embedded in each class and the discovery of hierarchical relations. Then, new network construction techniques will be explored to evaluate advancements in the structural network representation and its implications. Last but not least, the network representation and proper measures will be studied to assess and predict different levels of severity for each COVID-19 patient, which is critical for the prognosis of patients and important for optimizing hospital resources’ availability.
